# On the Personal Facets of Quality of Life in Chronic Neurological Disorders

**DOI:** 10.3233/BEN-2009-0243

**Published:** 2009-12-07

**Authors:** Anna R. Giovagnoli, Antonio Martins da Silva, Antonio Federico, Ferdinando Cornelio

**Affiliations:** ^1^Laboratory of Neuropsychology"C. Besta" Neurological InstituteMilanItaly; ^2^Department of NeurologyNeurosurgery and Behaviour SciencesUniversity of SienaSienaItaly; ^3^Neurological DepartmentUniversity of PortoPortoPortugal; ^4^Scientific Direction"C. Besta" Neurological InstituteMilanItaly

**Keywords:** Quality of life, brain lesions, chronic neurological disorders, mood, spirituality, cognitive functions

## Abstract

Quality of life (QOL) is an important clinical endpoint, but it remarkably varies in patients with similar neurological conditions. This study explored the role of spirituality (i.e., the complex of personal transcendence, connectedness, purpose, and values) in determining QOL in chronic neurological disorders.~Seventy-two patients with epilepsy, brain tumours or ischemic or immune-mediate brain damage compiled inventories for QOL (WHOQOL 100), spirituality (Spiritual, Religious and Personal Beliefs, WHOSRPB), depression (Beck Depression Inventory, BDI), anxiety (State-Trait Anxiety Inventory, STAI), and cognitive self-efficacy (Multiple Ability Self-Report Questionnaire, MASQ) and underwent neuropsychological testing. With respect to 45 healthy controls, the patients reported worse QOL, with no difference between the four patient subgroups. Factor analyses of the WHOSRPB, STAI, and BDI scores and of the MASQ and neuropsychological test scores yielded four (Personal Meaning, Inner Energy, Awe and Openness, Mood) and three factors (Control Functions, Cognition, Memory), respectively. Mood, Cognition, Inner Energy, schooling, and subjective health status correlated with the WHOQOL scores, but at regression analysis only Mood and Inner Energy predicted QOL. This suggests that spirituality, as a personal dimension distinct from mood, contributes to determine QOL. A multidimensional assessment of QOL, including personal facets, may explain differences between patients with chronic neurological disorders.

## 1. Introduction

In the biomedical field, different studies [5,13,19, 35–37] highlighted the subjective and multidimensional composition of health-related quality of life (QOL), contributing to develop theoretical definitions quite different from an initial conception of QOL as a mere index of physical autonomy [7]. In this regard, Calman [5] considered QOL as "the correspondence between hopes, expectations, ideals, and actual experiences", and Knippemberg and De Haes [36] comprehended "human values, the ability to manage own life, and the satisfaction of own necessities".

Many chronic neurological conditions (e.g. multiple sclerosis, epilepsy, brain tumours, vascular brain diseases) may cause severe impairments that reduce autonomy, change the future goals of patients and their families, and require effortful strategies to cope with the prospect of poor independence or short survival [15, 31]. Accordingly, in these patients, the subjective dimensions of QOL received increasing attention, becoming an important endpoint of clinical outcome together with disease-related aspects (e.g., disease free periods, specific clinical changes) [2,16,21]. Multiple cognitive, emotional, relational, and physical impairments were proven to determine QOL, but comparative studies inconsistently succeeded in explaining the variability of QOL in patients with similar neurologi-

ISSN 0953-4180/09/$17.00 © 2009 – IOS Press and the authors. All rights reserved cal or psychosocial conditions [8,18]. In this regard, Giovagnoli et al. [17] suggested that spirituality may contribute to determine QOL in epilepsy patients.

Spirituality is a complex dimension interfaced among the philosophical and health-related disciplines. In the psychosocial field, spirituality may be classified as a latent construct (i.e., a conceptual entity that cannot be directly observed) that is distinct from religiousness [25]. In relation to transcendence, spirituality is defined as the capacity to surpass physical, psychological, and social aspects of health and disease [1] and it is also distinguished into intra-personal (i.e., the potentiality of self) [14] and interpersonal spirituality (i.e., the connection with other people or superior entities) [10]. In relation to originality, major definitions deal with the complex of unique qualities of a person which give direction to the existence and meaning to human experiences [26], the dimension of life which gives cohesiveness to all the others [28] or the thing which gives motivation, energy and direction to a person's behaviour [12]. According to the World Health Organization (WHO) [38], spirituality includes different components, such as transcendence, connectedness, purpose, and values (e.g., faith, forgiveness, gratitude, sense of belonging, love), that are shared by people with different ethnic, cultural, and religious background.

In sum, while QOL has become an important indicator of outcome in many neurological conditions, its variability is only in part explained by disease-related, psychosocial or demographic aspects, suggesting the influence of yet unexplored personal variables. This study was carried out to verify the hypothesis that spirituality, as an independent personal dimension, contributes to determine the QOL of patients with chronic neurological disorders. Specific objectives were: a) to elaborate a multidimensional operational definition of QOL including different spiritual aspects distinct from mood, b) to compare QOL and spirituality in patients with different neurological disorders and healthy subjects, and c) to determine the relative contribution of spiritual, affective, socio-demographic, and disease-related variables in predicting patients' QOL.

## 2. Methods

### 2.1. Patients

Patients selection criteria were chronic neurological impairment, brain damage, and disease duration longer than one year. Accordingly, 72 patients (40 females, 32 males) with epilepsy ($n = 36$), brain tumours ($n = 15$), ischemic ($n = 14$) or immune-mediate brain damage ($n = 7$) were evaluated after informed consent. Patient characteristics were collected using a structured interview included in the WHOQOL 100 scale [37] that evaluates religion-related aspects (religiosity, belonging to a religious community, creed, personal beliefs) and health perception (very poor, poor, neither poor nor good, good, very good) using 5-point Likert scales, and socio-demographic information (age, gender, schooling, marital status, i.e., single, married, living as married, separated, divorced or widowed, the number of family members, work level, and financial level). Patient groups had different age (F $= 10.77$, $p < 0.001$) and education, as expressed by the years of schooling (F $= 4.50$, $p = 0.006$), due to younger age and higher education in the epilepsy ($p < 0.001$, $p = 0.004$) and brain tumour patients ($p = 0.03$, $p = 0.04$) with respect to the vascular patients. Disease duration was longer in the epilepsy patients with respect to the other patients but between-group differences were not significant (F $= 3.07$, $p = 0.04$). Forty-five healthy subjects (25 females, 20 females) were selected as controls among patients' relatives and the hospital staff if they were adult, had attended compulsory school and had similar social and economic background; they were as old as the brain tumour and immune-mediate brain damage patients but were older with respect to the epilepsy patients ($p = 0.03$) and younger with respect to the vascular patients ($p = 0.005$), (F $= 8.18$, $p < 0.001$), and reported more years of schooling than the vascular patients ($p = 0.001$), (F $= 4.44$ $p = 0.002$). The patients were similar to the controls in the other socio-demographic and religion-related aspects, but the patients with epilepsy or vascular or and immune-mediate disorders reported a significantly worse health status (F $= 12.68$, $p < 0.001$), (Table 1).

### 2.2. Self-evaluation instruments

Self-evaluation inventories for QOL (WHOQOL 100) [37], Spirituality (Spiritual, Religious and Personal Beliefs, WHOSRPB) [37], depression (Beck Depression Inventory) [17], anxiety (State-Trait Anxiety Inventory) [17], and Cognitive self-efficacy (Multiple Ability Self-Report Questionnaire) [33] were used.

The QOL instruments were chosen in relation to the lack of disease-specific items, previous standardization, documentation of adequate psychometric properties, and validation in ill and healthy people of different culture and ethnic origin [37]. The WHOS-

Table 1
Demographic, clinical, and religion and health-related aspects of neurological patients and healthy subjects

| | All patients ($n = 72$) | Epilepsy ($n = 36$) | Brain tumours ($n = 15$) | Vascular brain disorders ($n = 14$) | Immune-mediate brain disorders ($n = 7$) | Healthy subjects ($n = 45$) |
|---|---|---|---|---|---|---|
| Age | 44.90 ± 16.70 | 37.03 ± 15.61 | 46.80 ± 15.22 | 61.93 ± 9.30 | 47.29 ± 13.68 | 46.53 ± 15.68 |
| Schooling | 11.49 ± 4.01 | 12.44 ± 3.46 | 12.13 ± 4.45 | 8.21 ± 2.91 | 11.71 ± 4.92 | 12.67 ± 3.32 |
| Marital status | | | | | | |
| single | 22 | 15 | 4 | 3 | 0 | 15 |
| married | 45 | 20 | 9 | 9 | 7 | 27 |
| separated | 2 | 0 | 1 | 1 | 0 | 2 |
| widowed | 3 | 1 | 1 | 1 | 0 | 1 |
| Work level | | | | | | |
| paid/regular | 38 | 21 | 9 | 4 | 4 | 33 |
| unpaid/irregular | 34 | 15 | 6 | 10 | 3 | 12 |
| Financial level | | | | | | |
| low | 8 | 2 | 2 | 4 | 0 | 1 |
| average | 46 | 25 | 11 | 5 | 5 | 11 |
| high | 18 | 9 | 2 | 5 | 2 | 33 |
| Family members | | | | | | |
| 1–2 | 25 | 10 | 5 | 9 | 1 | 18 |
| 3–4 | 47 | 26 | 10 | 5 | 6 | 27 |
| Religiosity | 2.97 ± 0.99 | 2.88 ± 1.06 | 3.13 ± 0.74 | 3.07 ± 1.07 | 2.86 ± 1.07 | 2.91 ± 1.10 |
| Belonging to a religious community | 2.35 ± 1.10 | 2.17 ± 1.11 | 2.40 ± 1.12 | 2.43 ± 1.02 | 3.00 ± 1.15 | 2.67 ± 1.20 |
| Creed | 3.19 ± 1.04 | 3.19 ± 1.09 | 3.07 ± 0.88 | 3.21 ± 1.19 | 3.43 ± 0.97 | 3.53 ± 0.99 |
| Personal beliefs | 3.22 ± 1.05 | 3.17 ± 1.05 | 3.27 ± 1.03 | 3.14 ± 1.23 | 3.57 ± 0.78 | 3.69 ± 0.82 |
| Health perception | 2.94 ± 0.80 | 2.86 ± 0.68 | 3.27 ± 0.96 | 2.93 ± 0.91 | 2.71 ± 0.75 | 3.87 ± 0.54 |
| Disease duration | 7.04 ± 6.62 | 9.19 ± 8.26 | 4.47 ± 2.69 | 6.07 ± 4.04 | 3.43 ± 2.29 | |

RPB scale [37] includes 15 facets (Spiritual connection, Meaning and purpose in life, Experiences of awe and wonder, Wholeness and integration, Spiritual strength, Inner peace, Hope and optimism, Faith, Kindness to others, Love, Death and dying, Forgiveness, Acceptation, Ethic rules, and Inner independence). Each facets includes different questions whose answers are rated on a 5-point Likert scale where 1 indicates low, negative perceptions and 5 indicates high, positive perceptions. Fifteen facet scores were obtained calculating a mean item scores within each facet.

The WHOQOL 100 scale [37] consists of 25 facets each including four items. The facets are grouped into seven domains including different facets numbers [i.e., General QOL (single facet), Physical (pain and discomfort, energy and fatigue, sleep and rest), Psychological (positive feelings, thinking, learning, memory and concentration, self-esteem, bodily image and appearance, negative feelings), Level of independence (mobility, activities of daily living, dependence on medication or treatments, work capacity), Social relationships (personal relationships, social support, sexual activity), Environment (physical safety and security, home environment, financial resources, health and social care, i.e., accessibility and quality, opportunities for acquiring new information and skills, participation in and opportunities for recreation/leisure activities, physical environment, i.e., pollution, noise, traffic, climate, transport), and influence of personal beliefs (the role played by personal beliefs in one's own life). Individual items are rated on a 5-point Likert scale where 1 indicates low, negative perceptions and 5 indicates high, positive perceptions. The following scores were computed: the total WHOQOL 100 score (the sum of all item scores) and the domain scores (i.e., the mean of the facet scores within the domain multiplied by four, so that each domain score ranges between 4 and 20).

The BDI [17] total score (0–36) is the sum of all item scores (0–3) and is proportional to the level of depression. The STAI 1–2 [17] gives two total scores (20–80) that are the sums of all item scores (1–4) and are proportional to the level of actual and habitual anxiety, with higher scores indicating worse anxiety. The MASQ [33] includes 38 questions relative to the self-perception of five cognitive domains (i.e., language, visuo-perceptual abilities, verbal and visual memory, and attention/concentration); one to five points are attributed to each answer in relation to the frequency (almost always, often, sometimes, very seldom, almost never) of specific abilities or difficulties; the total score (38–190), sum of all subtest scores, is an index of everyday functioning: the higher the total score, the more serious the perceived difficulties.

Table 2
Mean WHOQOL scores in neurological patients and healthy subjects

| | All patients | Epilepsy | Brain tumours | Vascular brain disorders | Immune-mediate brain disorders | Healthy subjects |
|---|---|---|---|---|---|---|
| Total score | 345.83 ± 48.82 | 345.64 ± 41.45 | 352.53 ± 65.23 | 336.93 ± 52.94 | 350.29 ± 42.58 | 375.40 ± 38.41 |
| General QOL | 11.49 ± 4.01 | 12.08 ± 3.32 | 11.40 ± 5.06 | 9.21 ± 3.94 | 13.14 ± 4.02 | 12.67 ± 3.32 |
| Physical | 13.52 ± 2.66 | 13.88 ± 2.48 | 13.37 ± 3.09 | 12.83 ± 2.77 | 13.38 ± 2.67 | 15.19 ± 1.61 |
| Psychological | 13.37 ± 2.24 | 13.00 ± 2.07 | 13.78 ± 2.43 | 13.55 ± 2.52 | 14.00 ± 2.97 | 14.25 ± 2.08 |
| Level of independence | 13.86 ± 2.80 | 13.75 ± 2.41 | 14.70 ± 2.89 | 13.96 ± 3.45 | 12.29 ± 3.07 | 16.05 ± 1.68 |
| Social relationships | 14.07 ± 2.60 | 14.08 ± 2.25 | 14.33 ± 3.02 | 13.35 ± 3.08 | 14.90 ± 2.57 | 14.51 ± 2.24 |
| Environment | 14.16 ± 2.18 | 14.21 ± 2.13 | 14.30 ± 2.68 | 13.64 ± 2.19 | 14.66 ± 1.36 | 14.73 ± 1.83 |
| Influence of personal beliefs | 12.72 ± 2.00 | 12.62 ± 1.86 | 12.52 ± 2.62 | 12.78 ± 1.93 | 13.50 ± 2.53 | 13.46 ± 1.78 |

Table 3
Mean WHOSRPB, BDI, STAI, and MASQ scores in neurological patients and healthy subjects

| | All patients | Epilepsy | Brain tumours | Vascular brain disorders | Immune-mediate brain disorders | Healthy subjects |
|---|---|---|---|---|---|---|
| Spiritual connection | 2.51 ± 1.70 | 2.49 ± 1.03 | 3.73 ± 1.01 | 2.51 ± 1.10 | 2.10 ± 1.42 | 2.83 ± 1.25 |
| Meaning and purpose in life | 3.35 ± 0.81 | 3.20 ± 0.76 | 3.20 ± 0.91 | 3.64 ± 0.69 | 3.85 ± 0.86 | 3.66 ± 0.91 |
| Experiences of awe and wonder | 3.62 ± 0.76 | 3.46 ± 0.64 | 4.05 ± 0.84 | 3.50 ± 0.89 | 3.82 ± 0.64 | 3.82 ± 0.68 |
| Wholeness and integration | 3.25 ± 0.73 | 3.19 ± 0.71 | 3.05 ± 0.76 | 3.50 ± 0.69 | 3.54 ± 0.75 | 3.55 ± 0.56 |
| Spiritual strength | 2.70 ± 0.97 | 2.67 ± 1.03 | 2.65 ± 0.88 | 2.71 ± 0.84 | 3.00 ± 1.25 | 3.03 ± 0.76 |
| Hope and optimism | 2.95 ± 0.89 | 2.79 ± 0.86 | 3.08 ± 0.97 | 3.01 ± 0.85 | 3.39 ± 0.88 | 3.20 ± 0.78 |
| Faith | 2.71 ± 1.01 | 2.60 ± 1.04 | 2.82 ± 0.96 | 2.80 ± 0.94 | 2.86 ± 1.24 | 2.83 ± 1.05 |
| Kindness to others | 3.45 ± 0.77 | 3.43 ± 0.78 | 3.26 ± 0.85 | 3.46 ± 0.58 | 3.92 ± 0.87 | 3.68 ± 0.56 |
| Love | 3.19 ± 1.01 | 3.22 ± 0.99 | 3.20 ± 1.04 | 3.19 ± 1.14 | 3.03 ± 0.99 | 3.33 ± 0.87 |
| Inner peace | 2.94 ± 0.81 | 2.76 ± 0.81 | 3.02 ± 0.86 | 3.20 ± 0.70 | 3.21 ± 0.78 | 3.05 ± 0.77 |
| Death and dying | 2.91 ± 1.12 | 2.87 ± 1.14 | 2.75 ± 1.08 | 2.98 ± 1.11 | 3.35 ± 1.28 | 2.85 ± 1.02 |
| Forgiveness | 3.27 ± 0.66 | 3.26 ± 0.80 | 3.22 ± 0.37 | 3.13 ± 0.56 | 3.67 ± 0.40 | 3.23 ± 0.43 |
| Acceptation | 3.15 ± 0.61 | 3.10 ± 0.65 | 3.07 ± 0.75 | 3.18 ± 0.35 | 3.55 ± 0.39 | 3.89 ± 0.51 |
| Ethic rules | 3.72 ± 0.65 | 3.73 ± 0.83 | 3.73 ± 0.44 | 3.60 ± 0.29 | 3.87 ± 0.48 | 3.84 ± 0.48 |
| Inner independence | 3.40 ± 0.65 | 3.33 ± 0.67 | 3.44 ± 0.69 | 3.32 ± 0.62 | 3.79 ± 0.47 | 3.76 ± 0.52 |
| BDI | 7.19 ± 0.46 | 8.19 ± 6.65 | 6.17 ± 4.57 | 6.21 ± 6.88 | 1.67 ± 1.15 | 3.96 ± 3.72 |
| STAI1 | 44.42 ± 13.80 | 45.83 ± 14.21 | 41.33 ± 15.94 | 44.57 ± 12.98 | 33.00 ± 1.00 | 38.36 ± 11.89 |
| STAI2 | 44.98 ± 11.54 | 46.92 ± 11.84 | 41.00 ± 13.84 | 43.93 ± 9.38 | 34.67 ± 8.96 | 38.51 ± 10.18 |
| MASQ | 90.95 ± 28.14 | 94.50 ± 27.33 | 82.71 ± 28.31 | 90.36 ± 30.93 | 70.33 ± 23.75 | 70.93 ± 19.30 |

### 2.3. *Neuropsychological tests*

In the patient groups, standardized neuropsychological tests were used to assess selective (Attentive Matrices) and divided attention (Trail Making Test), reasoning (Raven's Coloured Progressive Matrices), planning (Tower of London) set-shifting (Wisconsin Card Sorting Test), comprehension (Token Test), Word Fluency on phonemic and semantic cues, visual perception (Street's Completion Test), constructive praxis (Rey's Complex Figure Copying), and short-term (Digit Span, Corsi's Blocks Span) and long-term memory (Short Story, Rey's Complex Figure Delayed Recall) [17]. Higher test scores indicate better performances except for the Trail Making test whose scores correspond to the seconds necessary to complete the trails.

### 2.4. *Data analysis*

The internal consistency of the WHOQOL 100 and WHOSRPB scales was assessed using Cronbach alpha. Factor analysis including the WHOSRPB facets, STAI1, STAI2, and BDI scores obtained by the neurological patients was used to determine the divergent validity of the spirituality and mood inventories. Individual scores were attributed to specific factors according to eigen values greater than 1 and factor loadings greater than 0.5. A separate factor analysis explored the distribution of the MASQ and neuropsychological test scores in order to summarize the components of cognitive functioning.

One-way ANOVA was used for comparing QOL and spirituality between the patient and control groups.

The determinants of patients' QOL were assessed by means of correlation, group comparison, and regression analyses. Pearson's product moment coefficients assessed the correlation of the WHOQOL 100 total and domain scores with the spiritual, mood-related, and cognitive factors (with significance level at p value $\leqslant 0.007$) and with age, education, the number of family members, subjective perception of

Table 4
Factor analysis of the WHOSRPB facets, STAI1, STAI2, and BDI scores in the neurological patients

| | Personal Meaning | Mood | Inner Energy | Awe and Openness |
|---|---|---|---|---|
| Spiritual connection | 0.77 | | | |
| Meaning and purpose in life | 0.65 | | | |
| Wholeness and integration | 0.75 | | | |
| Spiritual strength | 0.82 | | | |
| Faith | 0.87 | | | |
| Love | 0.78 | | | |
| Inner peace | | 0.69 | | |
| Hope and optimism | | 0.59 | | |
| BDI | | −0.69 | | |
| STAI1 | | −0.87 | | |
| STAI2 | | −0.87 | | |
| Forgiveness | | | 0.81 | |
| Ethic rules | | | 0.80 | |
| Inner independence | | | 0.70 | |
| Death and dying | | | 0.51 | |
| Acceptation | | | 0.54 | |
| Experiences of awe and wonder | | | | 0.67 |
| Kindness to others | | | | 0.74 |
| Total explained variance 66.36% | 22.46% | 18.71% | 14.92% | 10.27% |

STAI, State Trait Anxiety Inventory. BDI, Beck Depression Inventory.

health status, religiosity, and disease duration (with significance level at p value $\leqslant$ 0.01). T statistics and one-way ANOVAs compared the QOL scores in relation to gender, diagnosis, the level of work and financial resources, religion, and belonging to religious communities (with significance level at p value $\leqslant$ 0.01). Hierarchical regression analysis was used to assess the association between the WHOQOL 100 total scores and spirituality by controlling for modifiable (health-related, religion-related) and not modifiable variables (socio-demographic, disease-related). The socio-demographic (age, gender, schooling, marital status, the number of family members, work level, financial level), disease-related (diagnosis, disease duration), health-related (subjective perception of health status, mobility, and mood and cognitive factors), and religion-related variables (religiosity, belonging to a religious community, creed, personal beliefs) and the WHOSRPB factors were entered in five subsequent stages. Separate regression analyses explored the associations of the WHOQOL 100 domain scores.

## 3. Results

Tables 2 and 3 report the mean scale scores of the subject groups.

### 3.1. *Reliability and factor analyses of the scale and test scores*

The WHOQOL 100 (Cronbach alpha = 0.96) and WHOSRPB scale (Cronbach alpha = 0.94) showed high internal consistency. Factor analysis of the WHOSRPB, STAI1, STAI2, and BDI scores yielded four factors (Personal Meaning, Inner Energy, Awe and Openness, and Mood), (Table 4). Factor analysis of the MASQ and neuropsychological test scores (which were completed by 60 patients) produced three factors (Control functions, Cognition, Memory), (Table 5). With respect to the other patients, those who did not complete the neuropsychological tests (two with epilepsy, six with brain tumours, one with vascular damage, and three with immune-mediated disorders) had similar socio-demographic and religion-related characteristics, obtained similar WHOSRPB and WHOQOL total scores, and showed a slightly shorter disease duration ($t = -2.09$, $p = 0.042$), indicating that the second factor analysis reasonably reflected the situation of the whole patient group.

### 3.2. *Between-group comparisons of the scale scores*

Separate one-way ANOVAs comparing the patient and control groups showed significant differences in the WHOQOL 100 total score (F = 3.30, $p = 0.013$) and Physical (F = 5.06, $p = 0.001$) and Level of Independence domains (F = 5.5, p<0.001), BDI (F = 3.55, $p = 0.009$), STAI2 (F = 3.44, $p = 0.011$) and MASQ scores (F = 5.13, $p = 0.001$). With respect to the controls, the epilepsy patients showed lower WHOQOL 100 total and Level of Independence scores and worse scores in the depression, trait anxiety, and cognitive self-efficacy scales; the brain tumour lower scores

Table 5
Factor analysis of the neuropsychological test and MASQ scores in the neurological patients

| | Control Functions | Cognition | Memory |
|---|---|---|---|
| Trail Making Test A | −0.73 | | |
| Trail Making Test B | −0.72 | | |
| Tower of London | 0.68 | | |
| Phonemic Fluency | 0.84 | | |
| Semantic Fluency | 0.67 | | |
| Digit Span | 0.75 | | |
| Corsi Span | 0.68 | | |
| Raven's Coloured Progressive Matrices | | 0.56 | |
| Attentive Matrices | | 0.61 | |
| Wisconsin Card Sorting Test | | 0.62 | |
| Token Test | | 0.75 | |
| Street's Completion Test | | 0.73 | |
| Rey's Complex Figure Copying | | 0.89 | |
| Short Story | | | 0.92 |
| Rey's Complex Figure Delayed Recall | | | 0.68 |
| MASQ | | | 0.56 |
| Total explained variance 69.38% | 29.47% | 24.92% | 14.99% |

MASQ, Multiple Ability Self Report Questionnaire.

Table 6
Correlation analyses of the WHOQOL 100 scores with the mood, spiritual, and cognitive factors in chronic neurological patients

| | WHOQOL 100 total score | General QOL | Physical | Psychological | Level of independence | Social relationships | Environment | Influence of personal beliefs |
|---|---|---|---|---|---|---|---|---|
| Mood | $r = 0.63$, $p <0.001$ | $r = 0.67$, <0.001 | $r = 0.38$, $p = 0.003$ | $r = 0.69$ $p <0.001$ | $r = 0.38$ $p = 0.003$ | $r = 0.47$, $p < 0.001$ | $r = 0.46$, $p < 0.001$ | |
| Personal meaning | | $r = 0.27$, $p = 0.04$ | | | | | | |
| Inner energy | $r = 0.029$ $p = 0.002$ | | | $r = 0.27$, $p = 0.04$ | $r = 0.29$ $p = 0.025$ | | | $r = -0.45$, $p = 0.013$ |
| Control functions | $r = 0.42$ $p = 0.028$ | | | | | | | |
| Cognition | | | | | $r = 0.41$ $p = 0.033$ | | $r = 0.39$ $p = 0.042$ | $r = -0.51$, $p < 0.001$ |
| Memory | | $r = 0.39$, $p = 0.044$ | | | | | | |
| Subjective perception of health status | $r = 0.44$, $p < 0.001$ | $r = 0.46$ $p < 0.001$ | $r = 0.25$ $p = 0.031$ | $r = 0.39$ $p = 0.001$ | $r = 0.47$ p< 0.001 | $r = -0.28$ $p = 0.016$ | $r = -0.33$ $p = 0.004$ | $r = 0.38$ $p = 0.001$ |
| Age | $r = -0.29$, $p = 0.014$ | | $r = -0.27$ $p = 0.022$ | | | $r = -0.29$ $p = 0.012$ | $r = -0.27$ $p = 0.02$ | |
| Schooling | $r = -0.26$ $p = 0.03$ | | $r = 0.46$ $p < 0.001$ | | | | | |

in the Physical and Level of Independence domains; and the vascular patients lower scores in the Physical domain. No between-group differences were found in the WHOSRPB scale.

### 3.3. *Analyses of the determinants of QOL in the neurological patients*

QOL, as expressed by the WHOQOL 100 total score and domain scores, correlated with the Mood, Inner Energy, Personal Meaning, Control Functions, Cognition, and Memory factors. In addition, the WHOQOL 100 total scores correlated with the subjective perception of health status, age, and schooling (Table 6). No correlation was found with the Awe and Openness factor, the number of family members, religiosity, and disease duration. Between-group comparisons of the WHOQOL 100 total scores with respect to diagnosis and the other socio-demographic and religion-related variables did not show any differences.

At the first stage of hierarchical regression analysis, including the socio-demographic variables, age ($R^2 = 0.08$, F = 6.38, $p = 0.014$) and work level ($R^2 = 0.15$, F = 6.32, $p = 0.003$) explained 15% of the variance of the WHOQOL total scores. The disease-related variables did not show any effect. By entering the health-related variables, Mood explained 27% of the variance ($R^2$ = 0.27, F = 9.54, $p$ = 0.005). Among the religion-related variables, creed explained 9% of the variance ($R^2$ = 0.09, F = 6.81, $p$ = 0.011). Of the spiritual factors, Inner Energy ($R^2$ = 0.08, F = 5.12, $p$ = 0.027) explained 8% of the variance. When including the variables identified in a cumulative regression analysis, only Mood ($R^2$ = 0.40, F = 37.41, $p < 0.001$) and Inner Energy ($R^2$ = 0.48, F = 25.71, $p < 0.001$) explained 48% of the variance of the WHOQOL total scores.

Exploratory regression analyses revealed that the General QOL ($R^2$ = 0.33, F = 12.09 $p$ = 0.002), Psychological ($R^2$ = 0.34, F = 13.02 $p$ = 0.001), and Level of independence domain scores ($R^2$ = 0.23, F = 7.32 $p$ = 0.012) were predicted by the Mood factor, while the Environment ($R^2$ = 0.16, F = 4.58 $p$ = 0.04) and Influence of personal beliefs domain scores ($R^2$ = 0.20, F = 7.02 $p$ = 0.013) were predicted by the Cognition factor.

## 4. Discussion

The results of this study show that spirituality, as a distinct personal dimension, significantly contributes to predict QOL in patients with chronic neurological disorders.

According to our data, some spiritual factors, as expressed by the WHOSRPB questionnaire, which evaluates inner aspects irrespective from religiousness, are independent from anxiety and depression. Such result documents a divergent content validity between the scales that assess mood and spirituality, suggesting that distinct spiritual facets may characterize a person independently from affects or emotions. In particular, the Inner Energy factor (composed by forgiveness, ethic rules, inner independence, death and dying, and acceptation) reflects a subjective dimension of confidence and strength that maintains one's own sense of cohesion and stability and contributes to proper detachment and coping to life challenges. The Personal Meaning factor includes aspects (spiritual connection, meaning and purpose in life, wholeness and integration, spiritual strength, faith, and love) that give direction to behaviour and support personal transcendence with respect to sudden or unpleasant events, while the Awe and Openness factor (composed by experiences of awe and wonder and kindness to others) represents an ability to appreciate one's own and others' life and the external world with curiosity and availability.

In our study, the Mood, Cognition, and Inner Energy factors, schooling, and subjective perception of health status correlated with different WHOQOL scores, but hierarchical regression analysis showed that only Mood and Inner Energy significantly predicted QOL: Mood explained 40% and Inner Energy 8% of the variance of the WHOQOL total score. In addition, Cognition explained 16% and 20% of the variance of the Environment and Influence of personal beliefs domain, respectively. By contrast, patients with different neurological diagnosis reported similar QOL and, at regression analysis, diagnosis and disease duration showed no effect in predicting QOL. No socio-demographic or religion-related variable showed associations with the WHOQOL scores. These findings maintain that personal dimensions are fundamental components of subjective well-being in chronic neurological patients and may significantly determine QOL, surpassing the influence of other health-related and disease-related variables. The lack of large numbers of patients in each neurological subgroup did not allow to disentangle the position of particular impairments or disabilities to QOL. However, our data indicate that, with respect to healthy subjects, the epilepsy patients reported minor independence, greater depression and anxiety, and poorer cognitive self-efficacy, the brain tumour patients reported minor physical abilities and independence, and the vascular patients reported minor physical abilities, whereas the immune-mediate brain damage patients did not show any difference. On these grounds, it may be argued that chronic symptom burden, prognostic uncertainty, and the awareness of being affected by brain damage play a prominent role in the determination of QOL, although particular neurological symptoms (e.g., the unpredictability of seizures, the loss of mobility) may affect selective QOL dimensions in individual patients. To our knowledge, this is the first investigation that assesses the contribution of discrete spiritual factors in predicting QOL in neurological disorders by highlighting the fundamental role played by a combination of brain damage and chronic impairments. Previous studies usually neglected the position of spirituality to QOL [3,4,11,20,24,29,32]. Some studies reported on religious experiences [11], the neural substrates of mystical states [20,29,32], hyper-religiosity and mystic attraction in interictal states [20], and the personality of patients with temporal lobe epilepsy (characterized by religiosity, philosophical interests, and sense of personal destiny) [3], but the influence of spirituality on QOL was rarely approached [17]. The spirituality of patients with degenerative dementia was never specifically considered, although it was included in studies of mixed progressive life-limiting diseases treated by palliative care [24]. Coulson et al. [9] listed spirituality among the positive lifestyle components that contribute to modify the manifestations of vascular dementia. Taking into account the caregivers of dementia patients, Spurlock [34] claimed that their psychological burden is inversely related to their spirituality and suggested an incorporation of spiritual issues in intervention strategies, whereas Leblanc et al. [22] did not report any evidence that religiosity moderates caregivers' stress. In multiple sclerosis, spirituality was evaluated in the context of general studies including cancer and other chronic conditions [4], without considering specific disease-related aspects. As regards brain tumour patients, Osoba et al. [27] and Cella and Tulsky [6] inserted spirituality in different QOL scales but no study evaluated the interrelationships of spiritual well-being. A study [23] showed that amyotrophic lateral sclerosis patients and caregivers with higher spirituality have better QOL. Likewise, in not neurological conditions, previous investigations showed that spirituality and religious practices may contribute to physical health, contrast anxiety and depression [26], give meaning to illnesses [14], reduce the effects of serious symptoms on QOL [26] or improve QOL by enhancing the effects of rehabilitation in patients with chronic disturbances [30]. By extending previous information on the subject, our data also suggest that chronic neurological patients and healthy subjects share common spiritual facets, although we cannot exclude particular relationships in selective neurological pathologies. In this regard, limitations of the present study may be the lack of homogeneous patient numbers in each subgroup and the lack of a few neuropsychological data, although all of the patients completed the self-evaluations questionnaires in the lack of severe cognitive impediments.

To conclude, the results of our study support that spirituality could represent a missing element that contributes to explain differences in QOL between patients with chronic brain pathologies. Spiritual factors might influence a patient's perception of impairments and disabilities or their coping strategies and, consequently, their collaboration to examinations and treatments. There is indication for a new conceptualization of QOL in neurology. Taking into account the personal meaningfulness of disease and not just its functional outcome, a multidimensional approach might enlarge our view of health and healthcare. Further studies are needed to confirm our operational model in larger patient populations. In particular, a cross-cultural approach to such matter, thereby stimulating the interactions between biomedical and humanistic disciplines, could provide a validation of any results about the position of spirituality to QOL.

## Acknowledgements

The authors are grateful to the patients and healthy subjects who carefully participated to the study and to Chiara Paterlini who helped with scale scoring and database.
